# Measuring digital health literacy and its associations with determinants and health outcomes in 13 countries

**DOI:** 10.3389/fpubh.2025.1472706

**Published:** 2025-03-20

**Authors:** Diane Levin-Zamir, Stephan Van den Broucke, Éva Bíró, Henrik Bøggild, Lucy Bruton, Saskia Maria De Gani, Hanne Søberg Finbråten, Sarah Gibney, Robert Griebler, Lennert Griese, Øystein Guttersrud, Zuzana Klocháňová, Zdenek Kucera, Christopher Le, Thomas Link, Julien Mancini, Dominika Miksova, Doris Schaeffer, Carlota Ribeiro da Silva, Kristine Sørensen, Christa Straßmayr, Miguel Telo de Arriaga, Mitja Vrdelja, Jürgen Pelikan

**Affiliations:** ^1^School of Public Health, University of Haifa, Haifa, Israel; ^2^Department of Health Education and Promotion, Clalit Health Services, Tel Aviv, Israel; ^3^Psychological Sciences Research Institute, Université Catholique de Louvain, Louvain-la-Neuve, Belgium; ^4^Department of Public Health and Epidemiology, Faculty of Medicine, University of Debrecen, Debrecen, Hungary; ^5^Public Health and Epidemiology, Department of Health Science and Technology, Aalborg University, Aalborg, Denmark; ^6^Department of Health, Dublin, Ireland; ^7^Careum Foundation, Careum Center for Health Literacy, Zurich, Switzerland; ^8^Careum School of Health, Kalaidos University of Applied Sciences, Zurich, Switzerland; ^9^Department of Health and Nursing Sciences, Faculty of Social and Health Sciences, University of Inland Norway, Elverum, Norway; ^10^Competence Centre Health Promotion and Healthcare, Austrian National Public Health Institute, Vienna, Austria; ^11^School of Public Health, Bielefeld University, Bielefeld, Germany; ^12^Department of Nursing and Health Promotion, Faculty of Health Sciences, Oslo Metropolitan University, Oslo, Norway; ^13^The Norwegian Centre for Science Education Department, The Faculty of Mathematics and Natural Sciences, University of Oslo, Oslo, Norway; ^14^Department of Public Health, Faculty of Health Care and Social Work, Trnava University Trnava, Trnava, Czechia; ^15^Czech Health Literacy Institute, Prague, Czechia; ^16^Department of Community Health, The Norwegian Directorate of Health, Oslo, Norway; ^17^Department of Quality Measurement and Patient Survey, Austrian National Public Health Institute, Vienna, Austria; ^18^Aix Marseille University APHM INSERM, IRD, ISSPAM, SESSTIM, Cancer, Biomedicine & Society Group, Marseille, France; ^19^Direção-Geral da Saúde, Lisbon, Portugal; ^20^Global Health Literacy Academy, Risskov, Denmark; ^21^Católica Research Centre for Psychological, Family and Social Well-Being, Universidade Católica Portuguesa, Lisbon, Portugal; ^22^Communication Unit, National Institute of Public Health, Ljubljana, Slovenia

**Keywords:** digital health literacy, eHealth literacy, HLS_19_, digital health literacy measurement, measurement scale validation, health information technology literacy, M-POHL

## Abstract

**Introduction:**

Digital health information sources are playing an increasingly prominent role in health promotion, public health and in healthcare systems. Consequently, digital health literacy skills are likewise becoming increasingly important.

**Methods:**

Using a concept validation approach, the aim of the study was to validate the digital health literacy measure HLS_19_-DIGI, applied in the European Health Literacy Survey (2019–2021) of the WHO M-POHL network, analyzing data from 28,057 respondents from 13 countries. The instrument is a modified and extended version of the Digital Health Literacy Instrument (DHLI).

**Results:**

The scale displayed high internal consistency. Confirmatory factor analysis (CFA) strengthened the hypothesized one-factor structure. In most countries, the data displayed acceptable fit to the unidimensional Rasch partial credit model (PCM). Pearson correlation with a measure of general health literacy showed sufficient discriminant validity, and a social gradient was found. Testing for predictive validity showed that the scale score predicts health-related outcomes.

**Discussion:**

The study shows that considerable proportions of the general adult populations across countries in Europe have limited DHL skills. The level of DHL has direct potential consequences for some forms of health service utilization, in some countries. Implications of the study include recommendations for improving digital health literacy, promoting organizational health literacy and quality assurance for digital health information and resources.

## 1 Introduction

The increasing availability and use of health-related digital resources such as electronic health records, telehealth initiatives, digital health applications, and interactive communication options with public health agencies and health care providers (e.g., for making appointments or reporting medical/test results), places increasing demands on peoples’ abilities to use these applications and resources. Health care organizations and governments often promote or initiate and develop digital resources that require users to access, understand, appraise, and apply health information, as well as specific skills such as navigating digital health information. Simultaneously, the process of digitalization has enhanced the amount of online health information, as well as the number and variety of channels that are used for disseminating this information. More recently, artificial intelligence (AI) has entered the digital arena with tools such as ChatGPT and others. Due to the ubiquitous nature of digital communication, commercial companies and individuals are also seeking the public’s attention through digital channels, including social media. As a result, more interest-driven, manipulative, or simply false information is disseminated to the public, requiring particularly critical and analytical skills from the public and individual users (1).

It is therefore incumbent upon researchers, practitioners, and policy makers to recognize and realize the importance of understanding and improving people’s proficiency in using digital resources for managing their health conditions and for promoting their health. Concerns have been raised about whether increased digitalization of health services will help make health resources available for more people, or whether it will increase health inequalities by creating a digital divide (2). To answer this question, national population surveys should include validated measures of digital health literacy.

As a relatively new concept, digital health literacy (DHL) is distinct from digital literacy (3) in that it refers to a person’s specific ability to process health-related information and re-sources obtained from digital sources. It is also distinct from eHealth literacy. While eHealth and digital health are often used interchangeably and are closely related, eHealth focuses mainly on healthcare, while DHL is broader and also refers to mHealth (mobile), artificial intelligence (AI) and other emerging areas of innovation and information technology (4).

### 1.1 Existing research and measures of digital health literacy

The need to assess people’s use of electronic sources to access and process health-related information was first recognized by Norman and Skinner (5), who proposed the term eHealth literacy to refer to “the ability to seek, find, understand, appraise health information from electronic sources and apply the knowledge gained to addressing or solving a health problem.” In accordance with this definition, Norman and Skinner developed the eHealth Literacy Scale (eHEALS), a self-report measure assessing a person’s skills to find and evaluate health information on the Internet, which at the time was the primary online source for health information. Subsequently, eHealth literacy has been commonly assessed in health-related studies (6), and, together with media health literacy, has been conceptualized as the interplay of personal, situational, and contextual factors in the processing of health information from online sources and their interactions (2). A plethora of studies have examined media health literacy and eHealth literacy throughout the lifespan, from childhood and adolescence (7–9) through adulthood and among the older adult (10, 11). Additionally, it has been examined with respect to cultural transition (12), to specific health conditions (13), and to specific health behaviors (14), although studies on performance-based eHealth literacy are still scarce (15, 16). Empirically, general health literacy and eHealth literacy were found to be distinct but related concepts (17).

Yet, the rapid development of digital health sources beyond the Internet warrants new thinking about the definition and measurement of DHL regarding the use and application of digital health resources at the population level. While eHEALS has been used extensively to assess the self-reported consultation of online health information, the past decade has seen a proliferation of digital health information. This has broadened the potential for applying digital tools for health, but also the scope of digital skills needed to use these tools. These skills are at the core of the DHL concept. A recent study refers to DHL as the individual, social, technical, critical, and analytic skills that are required for searching, finding, understanding, evaluating, and applying digitally available health information (18). According to Bittlingmayer et al. (19), DHL refers not only to navigation in the digital space and to the use of digital information sources and their evaluation (e.g., health apps, social media, information sites on the Internet), but also to the individual “option spaces” that arise with increasing digitalization and the related availability of health information.

To capture these skills, a more comprehensive self-report scale (Digital Health Literacy Instrument- DHLI), which also addresses the interactive task of adding content, was developed, and validated in the Netherlands (16). While this scale captures various aspects of DHL, it has not been applied or field-tested in large samples, except for its use in an international cross-sectional survey among university students in the specific context of the COVID-19 pandemic (20).

### 1.2 Rational for a further developed digital health literacy measure

Valid and reliable knowledge about people’s DHL can help health authorities to discern the experiences and difficulties of patients and of the wider population, including the extent to which they perceive health information provided through digital sources as apparent, accessible, understandable, correct, and applicable. Yet, while DHL has been assessed in some countries in the European Region (21, 22), these assessments mainly used the eHEALS or similar instruments. As such, they have not looked at DHL in a broader societal context, nor provided relevant associations with other aspects of health literacy or examined DHL in a broader international context. Apart from national studies conducted as part of the M-POHL Health Literacy Survey 2019–2021 (HLS_19_) (23–25), no national health literacy survey to date has assessed and compared DHL to general health literacy on an international level. Moreover, there are no studies that have examined factors and covariates associated with DHL as determinants or its association with health promoting behavior, healthy lifestyles, early detection of diseases, use of health services, or self-care in the case of long-term illness.

The European Health Literacy Survey (HLS_19_) under the auspices of the WHO Action Network on Measuring Population and Organizational Health Literacy (M-POHL) (26) offered an opportunity to further develop and validate a further developed measure for DHL, to report on DHL at the general population level, and to study its association with general health literacy, with sociodemographic, socioeconomic, and other variables and with possible health-related consequences and outcomes.

This article is part of a series of papers, introducing health literacy tools that have been developed, applied and tested through the HLS_19_ study (27–30). The aim of this series is to use the data collected in the HLS_19_ to examine the psychometric properties of the newly or further developed health literacy tools and different aspects of their validity. In order to derive overarching and comparable conclusions about the HLS_19_ tools, the articles in this series address similar research questions and use the same analytical procedures.

## 2 Materials and methods

### 2.1 The HLS_19_-DIGI instrument

The DHL instrument further developed for the HLS_19_ survey, named HLS_19_-DIGI, is based on the DHLI measure (16) but aligned more strongly with the concept and model of general health literacy proposed by the HLS-EU study (27), and promoted by M-POHL. This model defines health literacy as the ability to access, understand, appraise, evaluate, and apply health information. Compared to the DHLI (16), the HLS_19_-DIGI adds the dimension of understanding digitally accessed health information and eliminates the redundancy on the topic of applying health information. In addition, the scope and diversity of digital health resources was broadened to include use and interaction with social media, health apps, wearables, personal health records, and interaction with healthcare providers. Thus, the HLS_19_ concept of DHL for promoting health includes the ability to search for, access, understand, appraise, validate, and apply online health information, and the ability to formulate and express questions, opinions, thoughts, or feelings when using digital devices.

**Figure 1:**
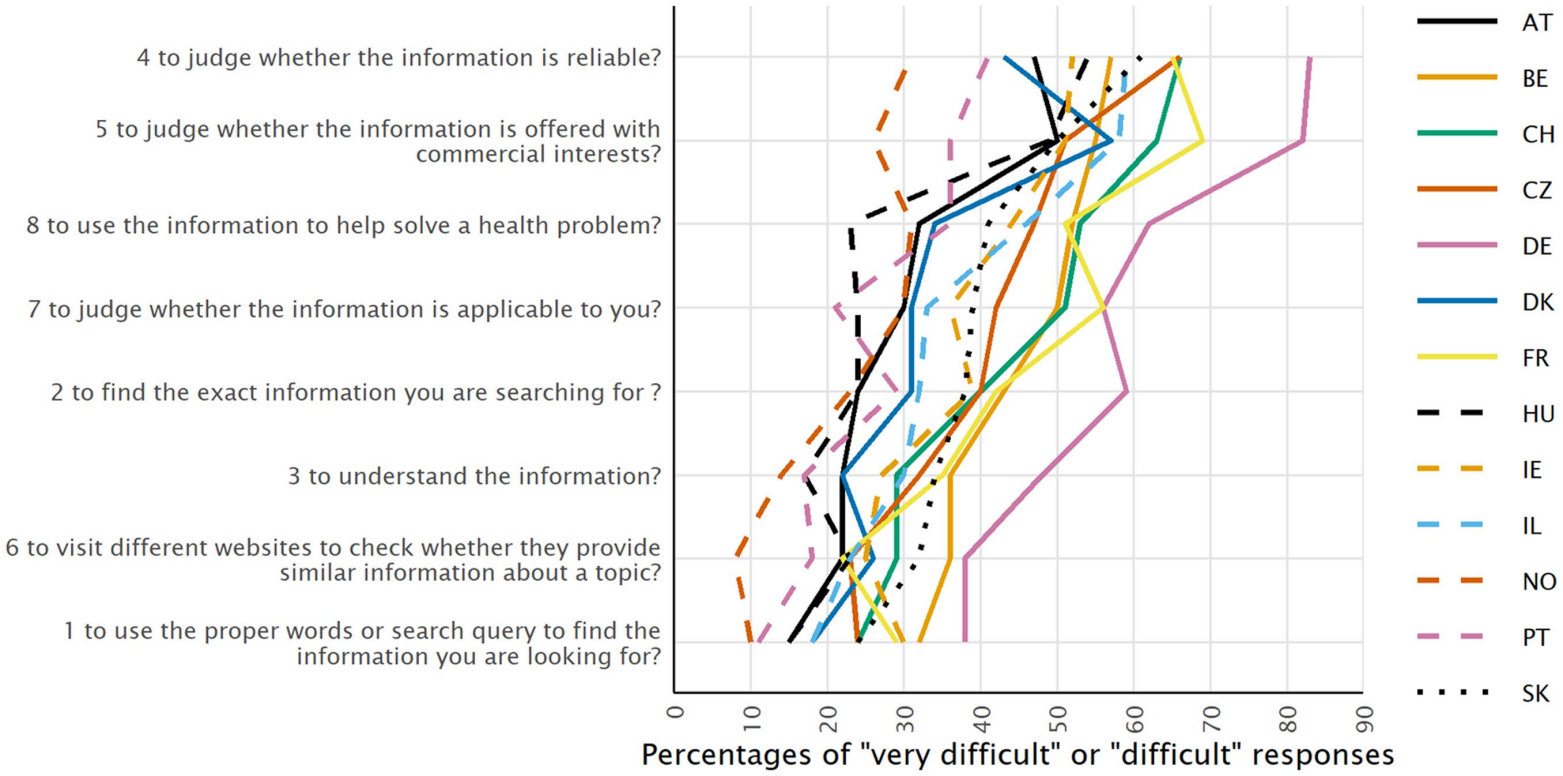
Percentage of respondents who responded "very difficult" or "difficult" to the items measuring the ability to access, understand, appraise and apply health information from digital sources (ranked by the overall percentage), for each country.

HLS_19_-DIGI consists of 10 items measuring two aspects of DHL: (1) the ability to search for, access, understand, appraise, and apply online health information measured by 8 items, namely HLS_19_-DIGI-HI e.g.: “How easy or difficult is it for you to use the right search terms and queries to find the health information you are looking for?,” or “…to judge whether the information you find online is reliable?”(Figure 1); and (2) the ability to clearly formulate questions, opinions, thoughts, or feelings when interacting by typing or posting information on a digital device (measured by 2 items, namely HLS_19_-DIGI-INT: “How easy or difficult is it for you to clearly formulate your written message when communicating with a health provider,” and “… to express your opinion, thoughts or feelings, ask a question in posting on social media including online forums”). In alignment with the format of other scales of the HLS_19_ survey, respondents are asked to rate each item on a four-point rating scale ranging from 4 = very easy to 1 = very difficult where a higher score indicates higher DHL.

Six additional items assess the frequency with which one uses various digital sources of health information, including websites, social media, digital devices related to health or health care, or digital interaction with the health system (such as online video consultations, digital personal health records and prescriptions, health related apps on mobile phone, etc.). For each of these sources, respondents are asked to indicate how many days, in a typical week, they use them for obtaining health-related information, with response categories ranging from “less than once per week,” “1–3 days per week,” “4–6 days per week,” “once a day,” to “more than once per day.” A mean score ranging from 1 (“less than once per week” or not relevant) to 5 (“more than once per day”) is calculated as a relative measure for the frequency of use of health-related digital resources.

We have adapted, shortened and extended the content of the DHLI tool (16), translated it into several languages and tested it, and this paper reports on the psychometric testing. The questionnaire was developed in English, and subsequently translated into 12 languages (Arabic, Czech, Danish, Dutch, French, German, Hebrew, Hungarian, Norwegian, Portuguese, Russian and Slovakian) by the HLS_19_ national teams and data collection agencies. Two forward translations were performed by 10 countries [Austria, Belgium (Dutch translation), Switzerland (German translation), Germany, Denmark, Hungary, Italy, Norway, and Slovakia], and one forward translation into Czech and Portuguese. Back translation was performed for five languages (Czech, Hebrew, Arabic, Russian and Norwegian). Countries with common languages collaborated in the translation process. For the German version, one forward translation was conducted by the national researchers of the Austrian, German and Swiss team, and one by the German national data collection agency, after which both versions were compared, and consensus was reached. For the French version, a translation into French was made by the language service of the Swiss Federal Office of Public Health (FOPH), reviewed by different experts, and consent reached between the Swiss, French and Belgian teams.

### 2.2 Data collection

Data on DHL were collected as national surveys for HLS_19_ in Austria (AT), Belgium (BE), Czech Republic (CZ), Denmark (DK), France (FR), Germany (DE), Hungary (HU), Ireland (IE), Israel (IL), Norway (NO), Portugal (PT), Slovakia (SK) and Switzerland (CH), resulting in a total of 28,057 respondents aged 18 and over, based on multi-stage random sampling or quota sampling procedures. Country specific sample sizes ranged from 1,000 to 4,487. Data collection involved a variety of methods and sampling procedures, including computer-assisted personal interviewing (CAPI), pen-and-paper personal interviewing (PAPI), computer-assisted telephone interviewing (CATI), and computer-assisted web interviewing (CAWI) (Table 1). Some countries applied different data collection methods for different subpopulations. Participation in a CAWI interview requires some familiarity with digital media and was used exclusively in BE, DK, and FR. In CH, CZ, and IL data were collected through CAWI for part of the sample, complemented by other methods.

**Table 1:** Data collection characteristics for digital health literacy by country.

Country	Languages	Type of data collection	Sampling procedure	Period of data collection	Valid responses
Austria	German	CATI	Multi-stage random sampling	16.03.2020–26.05.2020	2,967
Belgium	Dutch, French	CAWI	Quota sampling	30.01.2020–28.02.2020 and 01.10.2020–26.10.2020	1,000
Czech Republic	Czech	CATI, CAWI	Random digital procedure and random quota sampling	10.11.2020–24.11.2020	1,599
Denmark	Danish	CAWI	Multi-stage random sampling	11.12.2020–05.02.2021	3,602
France	French	CAWI	Quota sampling	08.01.2021–18.01.2021	1,000
Germany	German	PAPI	Multi-stage random and quota sampling	13.12.2019–27.01.2020	2,143
Hungary	Hungarian	CATI	Multi-stage random sampling	02.12.2020–20.12.2020	1,195
Ireland	English	CATI	Random digit dialing approach	24.07.2020–07.12.2020	4,487
Israel	Hebrew, Arab, Russian	CATI, CAWI	Multi-stage random sampling	15.12.2020–10.1.2021	1,315
Norway	Norwegian	CATI	Random sampling procedure within each stratum	04.04.2020–13.05.2020	2,855
Portugal	Portuguese	CATI	Random stratified sampling	10.12.2020–13.01.2021	1,247
Slovakia	Slovak	CAPI	Multi-stage random sampling	22.06.2020–14.09.2020	2,145
Switzerland	French, German, Italian	CAWIת CAWI	Multi-stage random sampling	05.03.2020–29.04.2020	2,502

The HLS_19_ study protocol required national samples to be weighted by gender, age groups, population density, and geographical areas/units, based on national census data to increase representativeness.

In addition to the 10 items of the HLS_19_-DIGI and the six additional items on the use of digital health information sources, data on the following sociodemographic and socioeconomic variables were collected: gender, age (in years), self-perceived social status (from 1 = “lowest self-perceived social status” to 10 = “highest self-perceived social status”) (27), the highest level of completed education (lower secondary or less: ISCED 0–2; higher secondary: ISCED 3; above secondary: ISCED 4–8), employment status (employed and unemployed or retired), and financial deprivation (“difficulty to pay all bills at the end of the month,” scored on a scale from 4 = “very easy” to 1 = “very difficult”) and whether or not the respondent has training in a health profession. To test for differential item functioning (DIF), we dichotomized variables on education level (ISCED 0–3 and 4–8) and social status (levels 1–4 and levels 5–10), and age categories were computed. More detailed information can be seen in Supplementary Table 1. Respondents were also asked to rate their self-reported general health on a scale ranging from very good to very bad, and to report limitations due to health problems (“severely limited”, “limited but not severely”, “not limited at all”), the existence of a long-term/chronic illness (lasting for 6 months or more: more than one, one, no) and their use of healthcare services (number of visits to GP/family doctor and number of specialist consultations in the past year) (13). General health literacy (GEN-HL) was measured, using the HLS_19_-Q12 self-assessment scale (26), which is a 12-item revised short version of the HLS-EU-Q47 (27), providing for a comprehensive operationalization of health literacy by measuring the perceived difficulty to access, understand, appraise and apply health information in three health domains (healthcare, prevention and health promotion). A four-point rating scale (4 = “very easy,” 3 = “easy,” 2 = “difficult,” and 1 = “very difficult”) was offered for the HLS_19_-Q12.

### 2.3 Data analysis

In a first step, perceived difficulties to access, understand, appraise, and apply online health information were analyzed for all countries by calculating the percentage of respondents who answered “very difficult” or “difficult” for each item. The scores of HL measures were calculated based on dichotomized as well as polytomous answer categories. To compensate for the potential resulting loss of information, item sets of categorical variables are also described by the average percentage response patterns (APRP), or the average percentage of how often a response category was selected within an item set. Given a data matrix consisting of m categorical items with k identical response categories for n respondents, an APRP is a compositional measure consisting of k percentages that describe for each of the k response categories how often the n respondents selected the respective category on average when answering the m categorical items (26). APRP were calculated separately for the eight items measuring the ability to access, understand, appraise, and apply online health information, and for the two items measuring the ability to interact with digital health resources.

Post-stratification weights were applied to the univariate, bivariate, and regression analyses described below. The base weights were calculated by the national teams and differ depending on the survey procedure. For country-by-country analyses, the effective weights were scaled so that their sum equals the number of valid cases in a country dataset. For analyses across all participating countries, the weights are rescaled so that the sum of weights by country equals 1,000, i.e., the countries have equal weight.

Next, scale scores measuring each of the two dimensions of the HLS_19_-DIGI were calculated by combining the respondents’ replies to the eight items related to accessing, understanding, appraising, and applying health information from digital sources HLS_19_-DIGI-HI, and separately the two items measuring the difficulty to interact with digital sources HLS_19_-DIGI-INT. Following Pelikan et al. (27), two types of scores are calculated and compared with respect to their performance. First, the “d-type score,” which is based on a count of the dichotomized items obtained by combining the “easy” and “very easy” and “difficult” and “very difficult” categories. The advantage of dichotomization is that it reduces the potential inequalities due to extreme responses in subpopulation which could benefit for example comparisons within countries or international comparisons, besides being easier to communicate the meaning of the score. Yet the disadvantage of dichotomization may be the loss of information. In order to address this issue, “p-type” scores were also calculated, as the mean of the numeric values ranging from 1 to 4. Both scores are scaled to the range of 0 to 100. The higher the score, the higher the level of DHL. Scores were computed only for respondents who had answered at least 80% of the items.

Spearman correlations were calculated among the eight items measuring the first DHL dimension (HLS_19_-DIGI-HI = i.e., the ability to search for, access, understand, appraise, and apply online health information), for the dichotomized items for all countries. Internal consistencies of the scales were tested for all countries using the Cronbach alpha coefficient, and the unidimensionality of the scales was tested using confirmative factor analysis (CFA) and Rasch analyses, based only on the polytomous items, respectively. For the latter, the data per country were tested against the unidimensional Rasch Partial Credit Model (PCM) (28). For each country, overall data-model fit, single item fit, the ordering of response categories, response dependency, one-dimensionality, and differential item functioning (DIF) were evaluated using an amended sample size of *n* = 480, corresponding to 20 respondents for each of 24 thresholds or *n* = 240 (10 respondents per threshold) for certain countries (28). These analyses were only performed for the first dimension (eight-item) of the tool (HLS_19_-DIGI-HI), as it would not be appropriate to perform them on the two-item scale measuring the difficulty of interacting with digital sources (HLS_19_-DIGI-INT). A detailed description of the methodological approach can be found in Griese et al. (29) and Finbraten et al. (30).

Finally, to investigate the determinants and health outcomes of DHL, the association of the HLS_19_-DIGI-HI subscale with sociodemographic and socioeconomic variables, general health literacy as measured by the HLS_19_-Q12, perceived health status and health care use was assessed by calculating Spearman’s *ρ* correlations and multiple linear regression analysis. Variables included in the regression models were: age, education (ISCED 0–8), self-perceived social status (1–10), financial deprivation, self-reported general health status, GP visits and specialist consultations.

## 3 Results

### 3.1 Use of digital information sources for health

Table 2 shows the proportion of responses, for all countries combined, regarding the use of different digital sources to obtain health information. Among those using digital resources for health, on average, websites are the most frequently consulted digital source on health information, followed by social media, digital interaction with the health system, a digital device related to health or health care, a health app on a mobile phone, and other.

**Table 2:** Use of different digital resources to obtain health information (% of respondents for all 13 countries combined).

Type of digital resources	Not relevant / do not know	< once per week	1–3 days per week	4–6 days per week	Daily	More than once per day
Internet (websites)	26.9	47.7	15.1	3.2	3.2	3.9
Social Media including online forums	42.6	39.3	9.0	2.6	3.1	3.4
A digital device related to health or health care	49.8	24.7	7.1	4.6	7.0	6.8
Health app on your mobile phone	52.6	24.8	7.2	3.9	7.0	4.5
Digital interaction with your health system	48.3	43.9	4.7	1.2	1.1	0.9
Other	70.7	24.2	2.2	1.0	0.9	0.9

### 3.2 Perceived difficulty to process digital health information

Figure 1 shows, for each item, the percentage of respondents who replied “very difficult” or “difficult” to the eight items measuring the ability to access, understand, appraise, and apply health information from digital sources, for each country (weighted equally). Percentage distributions for all four categories, for each item, are available in the Supplementary material of the International Report of the HLS_19_ Consortium (26). Although there is a considerable variation in perceived difficulty across countries, the ranking of the combined two difficulty response categories shows similar patterns across countries, with few exceptions. The percentages of combined “difficult” and “very difficult” responses range from 21.8% for item 1 (“to use the proper words or search query to find the information you are looking for”) to 54.1% for item 4 (“to judge whether the information is reliable”). For both items, the percentage of respondents finding these tasks (very) difficult is the lowest in Norway (9.5 and 30.6%) and highest in Germany (38.5 and 82.6%, respectively).

**Figure 2:**
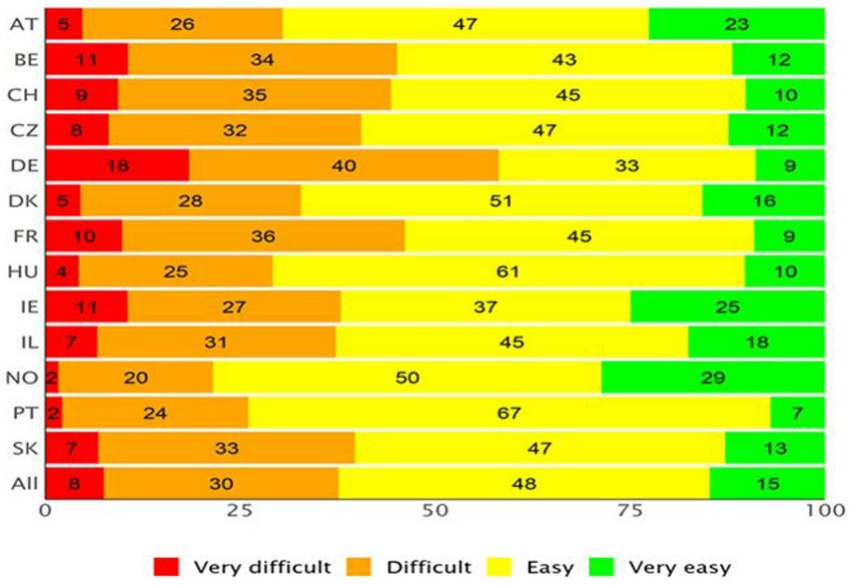
Average Percentage Response Patterns (APRP) for the item set measuring the ability to access, understand, appraise, and apply online health information, for each country (equally weighted).

**Figure 3:**
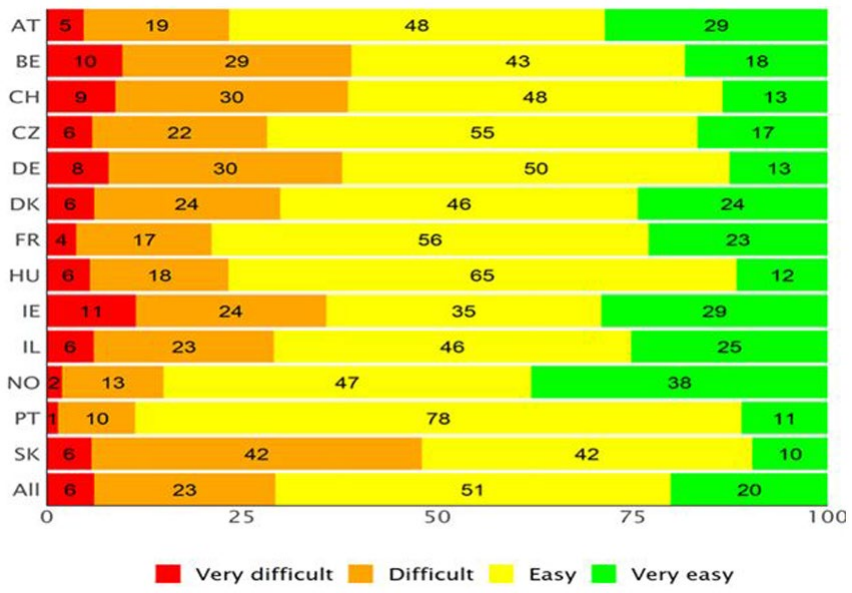
Average Percentage Response Patterns (APRP) for item set measuring the ability to interact with digital health devices, for each country (equally weighted).

Figures 2, 3 show the APRP for the two DHL dimensions of the HLS_19_-DIGI (i.e., the ability to access, understand, appraise, and apply online health information, and to interact with digital devices related to health, respectively) for each country. On average, nearly 40% of the respondents across all countries reported difficulties in processing digital health information (i.e., they responded “difficult” or “very difficult”), with Norway having the lowest proportion (22%) and Germany the highest (58%). Figure 3 shows that, on average, more than a quarter of respondents (29%) reported difficulties interacting with digital devices for health, but again, the numbers vary by country. Respondents from Portugal reported the lowest difficulties (11%) and respondents from Slovakia the highest (48%). In contrast, 51% of respondents experience interacting with digital devices for health as easy, with scores varying between 35% in Ireland and 78% in Portugal, and 20% as very easy, with scores varying from 10% in Slovakia to 38% in Norway.

### 3.3 Psychometric properties of the HLS_19_-DIGI scales

Spearman correlations among the eight items measuring the HLS_19_-DIGI-HI dimension of the HLS_19_-DIGI (i.e., the ability to access, understand, appraise, and apply online health information) ranged between 0.37 and 0.66 for all countries combined. The same levels of correlation are found for individual countries (26).

Cronbach’s alpha (*α*) for the HLS_19_-DIGI-HI items are above 0.70 for all countries, with an average α of 0.83 for the dichotomized items and 0.89 for the polytomous items (Table 3), indicating a reliable scale. The two HLS_19_-DIGI-INT items were highly correlated.

**Table 3:** Internal consistencies (Cronbach alpha) and fit indices for the one-factor confirmatory factor model for HL-DIGI-8 items, for each country and mean of all countries (equally weighted).

Fit index (Threshold value)	AT	BE	CH	CZ	DE	DK	FR	HU	IE	IL	NO	PT	SK	Mean
Dichotomized items														
Cronbach α	0.81	0.86	0.85	0.82	0.83	0.86	0.86	0.79	0.79	0.83	0.77	0.83	0.87	0.83
SRMSR	0.07	0.12	0.08	0.07	0.07	0.09	0.06	0.10	0.06	0.08	0.07	0.07	0.07	0.08
RMSEA	0.08	0.13	0.09	0.07	0.08	0.11	0.07	0.10	0.06	0.08	0.06	0.08	0.08	0.08
RMSEA (p)	0.00	0.00	0.00	0.00	0.00	0.00	0.00	0.00	0.06	0.00	0.05	0.00	0.00	0.01
CFI	0.98	0.98	0.99	0.99	0.99	0.98	0.99	0.96	0.99	0.99	0.98	0.99	0.99	0.98
TLI	0.97	0.97	0.98	0.98	0.98	0.98	0.99	0.94	0.98	0.98	0.98	0.98	0.99	0.98
GFI	0.98	0.97	0.99	0.98	0.99	0.98	0.99	0.97	0.99	0.99	0.99	0.99	0.99	0.98
AGFI	0.97	0.95	0.98	0.97	0.98	0.97	0.98	0.94	0.98	0.97	0.98	0.98	0.98	0.97
Polytomous items														
Cronbach α	0.89	0.91	0.91	0.88	0.91	0.92	0.89	0.84	0.86	0.89	0.87	0.89	0.92	0.89
SRMSR	0.05	0.09	0.07	0.06	0.06	0.07	0.06	0.10	0.04	0.07	0.06	0.05	0.05	0.06
RMSEA	0.10	0.18	0.14	0.10	0.11	0.15	0.12	0.17	0.07	0.12	0.11	0.10	0.12	0.12
RMSEA (p)	0.00	0.00	0.00	0.00	0.00	0.00	0.00	0.00	0.00	0.00	0.05	0.00	0.00	0.00
CFI	0.99	0.98	0.99	0.99	0.99	0.99	0.99	0.96	0.99	0.99	0.98	0.99	0.99	0.99
TLI	0.99	0.98	0.99	0.98	0.99	0.99	0.98	0.94	0.99	0.98	0.98	0.99	0.99	0.98
GFI	0.99	0.99	0.99	0.99	0.99	0.99	0.99	0.97	0.99	0.99	0.99	0.99	0.99	0.99
AGFI	0.98	0.96	0.98	0.98	0.98	0.98	0.98	0.93	0.98	0.97	0.97	0.99	0.99	0.97

AT, Austria; BE, Belgium; CH, Switzerland; CZ, Czech Republic; DE, Germany; DK, Denmark; FR, France; HU, Hungary; IE, Ireland; IL, Israel; NO, Norway; PT, Portugal; SK, Slovakia; Cronbach α (> 0.7); SRMSR (≤ 0.08), standardized root-mean square residual; RMSEA (≤ 0.06), root-mean-square error of approximation; CFI (≥ 0.95), comparative fit index; TLI (≥ 0.95).

On average, the HLS_19_-DIGI-HI and DIGI-INT scores are moderately correlated (with a Pearson correlation coefficient of *r* = 0.48 for the d-type score and *r* = 0.55 for the p-type score), but the level of correlation differs considerably across countries (from 0.21 to 0.60 for the d-type score and 0.24 to 0.68 for the p-type score).

Table 3 shows the fit indices for the single-factor confirmatory factor model for the HLS_19_-DIGI-HI scale. The indices generally indicate an acceptable fit for all countries, although the standardized root mean square residual (SRMSR, assuming a 0.08 threshold value) is too high in three countries and the lower bound of the confidence interval of the root mean square error of approximation (RMSEA, with 0.05 as threshold) is too high in all countries, suggesting a possible misfit between the observed data and the single-factor model. However, the other fit indices (comparative fit index, Tucker-Lewis index, goodness of fit and adjusted goodness of fit index) overall indicate a sufficient fit between the observed covariance matrix and the model implied covariance matrix for all countries.

Using an amended sample size of *n* = 480 the data showed an overall sufficient fit to the Rasch model (χ^2^ statistic) for data collected in Austria, Germany, Ireland, Norway, and Switzerland. We observed acceptable fit for the data collected in Belgium and in the Czech Republic, and, with a further reduced sample size of *n* = 240, also for the data collected in Denmark, Hungary, Israel, and Portugal. A principal component analysis (PCA) of Rasch model residuals, combined with dependent t-tests to identify possible subscales, revealed that the HLS_19_-DIGI-HI scale could be considered sufficiently unidimensional for most countries. The HLS_19_-DIGI-HI scale was also considered well-targeted in most countries. The thresholds, and thus the response categories, were ordered and functioned well.

There is no significant statistical dependency between pairs of items, which means that no items are “too similar” and therefore redundant. However, item 5 “to judge whether health-related information is offered with commercial interests” tends to under-discriminate in most countries and thus does not strictly correspond to the latent trait that is underlying the scale. Some items displayed DIF for person factors, such as age and gender, but there was no consistent pattern across countries. Furthermore, the scale did not measure invariantly across countries, as the item threshold locations or item “difficulty order” varied between countries. The latter may be ascribed to DIF for country or language.

### 3.4 Content and concurrent validity of the HLS_19_-DIGI scales

Both scales of the HLS_19_-DIGI are supported by a theory-based model that justifies the selection of items for the measures. Table 4 shows the distribution of the DHL score measuring the ability to access, understand, appraise, and apply digital health information for each country. For the d-type score, the mean score across all countries (equally weighted) is 62.3 (on a scale from 0 to 100), varying between 41.8 (DE) and 78.7 (NO). For most countries, the distribution is left skewed with the 75% percentile starting at the maximum value, indicating a ceiling effect for the measure. For the p-type score, the mean score across all countries (equally weighted) is 56.6 (on a scale from 0–100), varying between 44.1 (DE) and 68.6 (NO). The p-scores are less skewed than the d-scores.

**Table 4:** Mean, median, quartiles, and standard deviation of the HLS_19_-DIGI-HI score, for each country and for all countries (equally weighted).

	Mean	SD	25th percentile	Median	75th percentile
Dichotomized items					
AT	70.1	29.2	50.0	75.0	100.0
BE	54.9	34.6	25.0	50.0	87.5
CH	55.7	33.0	25.0	62.5	87.5
CZ	59.3	31.4	37.5	62.5	87.5
DE	41.8	31.6	12.5	37.5	62.5
DK	67.2	32.2	37.5	75.0	100.0
FR	53.9	33.5	25.0	50.0	87.5
HU	71.8	26.9	62.5	75.0	100.0
IE	62.1	30.2	37.5	62.5	87.5
IL	62.7	31.0	37.5	62.5	87.5
NO	78.7	24.7	62.5	87.5	100.0
PT	74.0	28.7	57.1	85.7	100.0
SK	60.3	34.2	37.5	62.5	100.0
All	62.3	32.5	37.5	62.5	100.0
Polytomous items					
AT	62.9	19.7	50.0	62.5	76.2
BE	52.0	21.2	37.5	54.2	66.7
CH	52.3	20.0	41.7	54.2	66.7
CZ	54.6	18.7	41.7	54.2	66.7
DE	44.1	21.6	33.3	45.8	58.3
DK	59.5	19.4	45.8	58.3	70.8
FR	51.0	18.9	37.5	50.0	66.7
HU	59.6	14.7	54.2	62.5	66.7
IE	58.9	21.9	45.8	58.3	75.0
IL	57.8	19.7	45.8	58.3	66.7
NO	68.6	17.0	58.3	66.7	79.2
PT	59.6	14.5	50.0	62.5	66.7
SK	55.4	20.6	41.7	54.2	66.7
All	56.6	20.2	45.8	58.3	66.7

AT, Austria; BE, Belgium; CH, Switzerland; CZ, Czech Republic; DE, Germany; DK, Denmark; FR, France; HU, Hungary; IE, Ireland; IL, Israel; NO, Norway; PT, Portugal; SK, Slovakia.

In terms of concurrent validity, Pearson correlations between the ability to access, understand, appraise, and apply digital health information measured by the HLS_19_-DIGI-HI and general health literacy measured by the HLS_19_-Q12 varies, for the d-type scores, between 0.44 (BE) and 0.67 (IL), with a mean of 0.53. For the p-type scores, the correlation coefficients range from 0.52 (BE, CH, HU) to 0.72 (IL) with a mean of 0.58. This confirms the assumption that both concepts are strongly related, but also sufficiently distinct to warrant separate measures. Across countries, the Spearman correlation between the scale and a score for the use of digital resources is *ρ* = 0.15, ranging from 0.04 (for BE) to 0.31 (for DE). So, for most countries, the perceived ease of using digital health information sources is moderately related to the use of such sources.

### 3.5 Determinants of DHL

Correlational analyses indicate that in most HLS_19_ countries the score on the HLS_19_-DIGI-HI scale measuring the ability to access, understand, appraise and apply digital health information is moderately correlated with having had training in a health profession (Spearman *ρ* = 0.14 for all countries, varying between 0.03 for AT and 0.18 for SK). The same applies to self-perceived social status in society (*ρ* = 0.13, ranging from 0.00 in AT to 0.25 in SK), age (*ρ* = −0.11, ranging from 0.03 in IL to −0.25 in DE), and education level (*ρ* = 0.07, varying from 0.03 in the CZ to 0.31 in SK). The relationship with education is more or less linear, meaning that the score on the HLS_19_-DIGI-HI scale increases with educational level. In contrast, the correlation with gender (overall *ρ* = 0.01) is very low for all countries.

Supplementary Table 2 shows the deviation from the population mean d-score for the scale measuring the ability to access, understand, appraise, and apply digital health information for selected subpopulations that are potentially vulnerable or disadvantaged regarding digital health literacy. Notably, older people show, on average, the largest deviation from the general population (−12), followed by those with a (very) bad self-perceived health (−11.4), financial deprivation (−8.7), low education (−7.8), and low social status (−6.8). Those who often visit their GP/family doctor also score markedly lower (−6.9). A somewhat lower level of deviation from the general population score is seen for people with limitations due to health problems (−4.7) and for those suffering from a long-term/chronic illness (−3.2). For most subpopulations the variation between countries is considerable.

To identify possible associations among the determinants of DHL, a series of regression models (Supplementary Table 3) were tested with the 8-item HLS_19_-DIGI-HI scale as the dependent variable. A first model including the core social variables of age, gender, education level, self-perceived social status and financial deprivation as independent variables explains 6% of the variance in DHL for all countries weighted equally, with the explained variance varying from 2% (BE) to 23% (SK). The best predictor of HLS_19_-DIGI-HI for all countries is financial deprivation (overall ß = −0.15; significant for 11 countries with ß values between −0.08 and − 0.27), followed by age (overall ß = −0.13; significant for 6 countries with ß values between −0.15 and − 0.26) and self-perceived social status (overall ß = 0.08; significant for 10 countries with ß values between 0.05 and 0.13). Education and gender have, on average, a smaller effect on the level of DHL, with lower overall ß values (0.03) and a significant effect in only a few countries. As such, the results suggest a social gradient for DHL, albeit with an inconsistent pattern across countries. A second model (not shown in the tables), in which use of digital resources is added as an independent variable, gives a small increase of the explained variance to 7% (ranging between 2% in BE and 24% in SK). Again, financial deprivation (overall ß = −0.14), age (overall ß = −0.12) and social status (overall ß = 0.08) are the strongest predictors, along with use of digital sources (overall ß = 0.11), while gender and education level have little influence (overall ß = 0.02).

### 3.6 Outcomes of DHL

The importance of DHL for health can be inferred from its relationship with a number of health outcomes. Significant Spearman correlations are found between the scores on the HLS_19_-DIGI-HI scale measuring the ability to access, understand, appraise, and apply digital health information and self-perceived health (*ρ* = −0.18 for all countries, with ρ varying between −0.10 in IE and − 0.28 for DE), limitations due to illness or health problems (*ρ* = 0.18 for all countries), and long-term illness or health problems (*ρ* = −0.11 for all countries).

The contribution of DHL to health outcomes can be derived from a series of multiple regression analyses using the HLS_19_-DIGI-HI score in combination with gender, age, education, self-perceived social status, and financial deprivation as independent variables (Supplementary Table 4). When self-perceived health is entered as the outcome variable, the model explains 15% of the variance for all countries combined (R^2^ ranging between 10% in IE and 26% in SK). Although age (overall ß = 0.24, ranging from 0.07 to 0.38, significant for all countries except BE) and self-perceived social status (overall ß = −0.17, ranging from −0.29 to −0.09, significant for all countries) are stronger predictors of self-perceived health, DHL also significantly contributes to the regression in nine out of the 13 countries participating in the study. The overall ß = −0.10, with ß values for individual countries ranging from −0.05 to −0.14.

However, when more objective outcome measures are considered such as the reported number of GP/family doctor visits or specialist consultations, DHL becomes less important as a determinant. For GP/family doctor’s visits, the model explains 6% of the variance for all countries combined (R^2^ ranging between 4% in IL and 14% in DE). On par with financial deprivation (average ß = 0.08, significant for 10 countries with ß between −0.01 and 0.17), DHL is the third most important contributor to the regression model, with ß values ranging from −0.01 to −0.10 (overall ß = −0.08). For specialist consultations, the variance explained by the model is only 5% (R^2^ ranging between 1 and 11%), and DHL’s contribution to the regression is only significant in SK (ß = −0.08) and an overall ß of −0.07. Thus, the level of DHL has direct potential consequences for some forms of health service utilization, in some countries.

## 4 Discussion

DHL is gaining momentum in public health and health service research, policy and practice. The rapid development of digital health information sources and resources beyond the Internet has widened the potential for the digitalization of health services, but also created a need to address the necessary digital skills of individuals to use these resources. Aligned with these developments, DHL refers to the individual, social, technical, critical, and analytic skills that a person needs in order to find, access, understand, evaluate, and apply digitally available health information (18). While existing research on DHL tends to take a narrow view of the topic, limiting it to finding and evaluating health-related information on the Internet, we detected a need to examine the subject more broadly in terms of the skills needed to navigate the digital space, access and use digital information sources such as health apps including wearables, social media, information websites, evaluate them, and take decisions on one’s health based on the options offered. A systematic bibliometric analysis on DHL did not take into consideration the validation of psychometrics and the sub-skills embedded in the term DHL (31).

This article described the conceptual background, development, and validation of a further developed instrument to measure DHL at the population level and investigated its determinants and associations with health outcomes. The instrument, named HLS_19_-DIGI, builds on the conceptual model, definition and dimensions of general health literacy proposed by the HLS-EU consortium (32) to measure the perceived ability of respondents to find, understand, appraise, and apply specifically digitally available health related information, and interact with digital information sources. Its validation is based on data of the European Health Literacy Survey 2019–2021 (HLS_19_) undertaken by the WHO Action Network on Measuring Population and Organizational Health Literacy (M-POHL) (26), involving approximately 28,000 participants in 13 countries in measuring DHL.

The instrument consists of 10 items measuring two aspects of DHL: the ability to access, understand, appraise/validate, and apply online health information (HLS_19_-DIGI-HI), and the ability to clearly formulate questions, opinions, thoughts, or feelings when interacting by typing or posting information on a digital device (HLS_19_-DIGI-INT). With regard to the first aspect, our results demonstrate that the 8-item scale measuring this dimension is sufficiently robust and valid. The internal consistency, confirmatory factor analysis and Rasch analysis confirmed its unidimensional structure for the data of all countries. The reliability of the scale, based on Cronbach’s alpha, is sufficiently high, with values ranging from 0.77–0.87 for the d-score and 0.84–0.92 for the p-score. The thresholds, and thus the response categories, are ordered and well-functioning. The correlation with general health literacy is high but not extreme (*r* = 0.55 for p-type scores and 0.48 for d-type scores), suggesting that both scales measure parts of the same construct, but are independent enough to be treated as different entities. One item tends to discriminate somewhat poorly across countries and some items display DIF for country or language, which could limit international comparisons. Future research could investigate whether minor changes to items like, e.g., 6 “to visit different websites to check whether they provide similar information about a topic” or 5 “to judge whether the information is offered with commercial interests” (Figure 1) could improve the model fit. Also, in some countries, based on the d-type score the scale displays a ceiling effect, with more than 25% scoring the highest value.

Regarding the second component (HLS_19_-DIGI-INT), the two items measuring difficulties in clearly formulating questions, opinions, thoughts or feelings when posting information on a digital device, are highly correlated, and, in some countries, do not differentiate well, suggesting that the items should be adjusted. The two items are, on average, moderately correlated (d-type score *r* = 0.48 and p-type score *r* = 0.55), but this differs considerably between countries. In the next M-POHL Health Literacy Survey 2024–2026 (HLS_24_), a refined measure may be considered for this aspect of DHL.

Using the HLS_19_-DIGI-HI, it was possible to document the perceived difficulty to process digital health information at the population level. Thus, it was seen that a significant part of the population – between 22 and 58%, depending on the country – finds it difficult to find, access, understand, evaluate, and apply digitally available health information. On average, 8% of the respondents (percentages ranging from 2 to 18% for the different countries) found it very difficult, and another 25% found it difficult (percentages ranging from 20 to 40%).

The most difficult tasks are to judge/appraise whether the information is offered with commercial interests (item 5), or whether information is reliable (item 4). Using proper words or search queries to find information one is seeking or visiting different websites to check whether they provide similar information is considered less difficult. This resonates with results from other (health) literacy research that with the ubiquitous stream of information in today’s society, the challenge is less to find information, but rather to discern whether it is correct, complete, and useful, aligned with the concept of critical health literacy (33).

In terms of its determinants, a social gradient for DHL is seen for all countries, but to a considerably different degree for different countries and indicators. The strongest social predictors of DHL are, on average, financial deprivation, age, and self-perceived social status in society. Perceived social status has acknowledged as a contribution to other aspects in a population’s social gradient (34). Education and gender are less significant, and only in some countries. Regarding education, ISCED levels were handled as a continuous variable. Different ISCED levels refer to different intervals of “years of education,” and these intervals also differ somewhat across countries. Thus, in future studies the measure may best be continuous such as number of years in an educational setting. General health literacy is a very strong predictor of DHL for all countries, and use of digital resources for most countries. Older people, people with poor health, and people with a low education level are most probable to have low DHL skills, which confirms the findings of other studies (16). This finding supports the conclusion of studies that have stressed the importance of interventions that address DHL in socially disadvantaged groups (35).

Regarding its importance for health and health care use, it was seen that DHL correlates with self-perceived health and limitations due to long-term illness, and that in most countries it is a determinant of self-perceived health along with age and self-perceived social status, but that its impact on healthcare use (i.e., GP visits and specialist consultations) is generally less strong.

Future analyses based on the study can examine the association between DHL and health behavior indicators measured in HLS_19_ (smoking, physical activity, BMI, alcohol, fruit and vegetable consumption).

### 4.1 Limitations

Differences in sampling and data collection limit the extent of country comparisons. Web-based surveys may have biased the results toward over-reporting of high DHL. For some countries, the non-response rates for several items were markedly higher than for other HLS_19_ measures, which may be partly due to the fact that people cannot evaluate something they do not do in everyday life, namely if they do not use the Internet to search for health information. As a result, the results may not be representative of all country samples. For example, in Ireland (IE), the group with missing data have a higher mean age than the total sample and a lower mean level of education. Furthermore, some of the countries collected data prior to the COVID-19 pandemic, others during the pandemic and some following the major pandemic waves. This diversity may reduce the extent to which participants in different countries can be compared, since the pandemic may have influenced the extent to which populations became digitally health literate. Yet, the measures across countries seems to behave rather uniformly, despite different data collecting modes, and the general picture of associations between determinants and DHL and with outcome measures also suggest that our conclusions are well supported. As is the case in all cross-sectional surveys association between variables does not infer causation. The next cycle of the M-POHL HLS survey can consider ways to mitigate these limitations.

## 5 Conclusions and implications

A compact, conceptually sound instrument to measure DHL was validated for 13 languages in 13 countries, showing acceptable psychometric properties. The study shows that considerable proportions of the general adult populations across 13 countries in Europe have limited DHL skills, that the level of DHL shows a social gradient, and that it is associated with indicators of health status. Professionals and policy makers in public health, health promotion and healthcare services should recognize the difficulties that large groups of society, especially the most vulnerable, have in accessing, understanding, appraising, validating and applying health information from digital sources and interacting with digital services. This challenge will become more critical as healthcare systems continue to transition into digital avenues of communication and information sharing with patients and communities (36). Addressing these difficulties should include organizational health literacy (OHL) action such as quality assurance of digital health resources (37), promoting critical DHL skills and expanding research to include populations from all ages in the life course. Finally, as artificial intelligence emerges and becomes more accessible for use in healthcare and public health (38), DHL skills will become increasingly important among the population-at-large to leave no one behind.

## Data Availability

The datasets presented in this study can be found in online repositories. The names of the repository/repositories and accession number(s) can be found at: https://m-pohl.net/Design_Methods.

## Author’s note

The authors are part of the WHO Action Network on Measuring Population and Organizational Health Literacy (M-POHL Network). The M-POHL Network is initiator of the HLS_19_.
