# Correction: Measuring digital health literacy and its associations with determinants and health outcomes in 13 countries

**DOI:** 10.3389/fpubh.2025.1655721

**Published:** 2025-08-06

**Authors:** Diane Levin-Zamir, Stephan Van den Broucke, Éva Bíró, Henrik Bøggild, Lucy Bruton, Saskia Maria De Gani, Hanne Søberg Finbråten, Sarah Gibney, Robert Griebler, Lennert Griese, Øystein Guttersrud, Zuzana Klocháňová, Zdenek Kucera, Christopher Le, Thomas Link, Julien Mancini, Dominika Miksova, Doris Schaeffer, Carlota Ribeiro da Silva, Kristine Sørensen, Christa Straßmayr, Miguel Telo de Arriaga, Mitja Vrdelja, Jürgen Pelikan

**Affiliations:** ^1^School of Public Health, University of Haifa, Haifa, Israel; ^2^Department of Health Education and Promotion, Clalit Health Services, Tel Aviv, Israel; ^3^Psychological Sciences Research Institute, Université Catholique de Louvain, Louvain-la-Neuve, Belgium; ^4^Department of Public Health and Epidemiology, Faculty of Medicine, University of Debrecen, Debrecen, Hungary; ^5^Public Health and Epidemiology, Department of Health Science and Technology, Aalborg University, Aalborg, Denmark; ^6^Department of Health, Dublin, Ireland; ^7^Careum Foundation, Careum Center for Health Literacy, Zurich, Switzerland; ^8^Careum School of Health, Kalaidos University of Applied Sciences, Zurich, Switzerland; ^9^Department of Health and Nursing Sciences, Faculty of Social and Health Sciences, University of Inland Norway, Elverum, Norway; ^10^Competence Centre Health Promotion and Healthcare, Austrian National Public Health Institute, Vienna, Austria; ^11^School of Public Health, Bielefeld University, Bielefeld, Germany; ^12^Department of Nursing and Health Promotion, Faculty of Health Sciences, Oslo Metropolitan University, Oslo, Norway; ^13^The Norwegian Centre for Science Education Department, The Faculty of Mathematics and Natural Sciences, University of Oslo, Oslo, Norway; ^14^Department of Public Health, Faculty of Health Care and Social Work, Trnava University Trnava, Trnava, Czechia; ^15^Czech Health Literacy Institute, Prague, Czechia; ^16^Department of Community Health, The Norwegian Directorate of Health, Oslo, Norway; ^17^Department of Quality Measurement and Patient Survey, Austrian National Public Health Institute, Vienna, Austria; ^18^Aix Marseille University APHM INSERM, IRD, ISSPAM, SESSTIM, Cancer, Biomedicine & Society Group, Marseille, France; ^19^Direção-Geral da Saúde, Lisbon, Portugal; ^20^Global Health Literacy Academy, Risskov, Denmark; ^21^Católica Research Centre for Psychological, Family and Social Well-Being, Universidade Católica Portuguesa, Lisbon, Portugal; ^22^Communication Unit, National Institute of Public Health, Ljubljana, Slovenia

**Keywords:** digital health literacy, eHealth literacy, HLS_19_, digital health literacy measurement, measurement scale validation, health information technology literacy, M-POHL

In the published article, the article title was “HLS_19_-DIGI - a new instrument for measuring digital health literacy: development, validation and associations with determinants and health outcomes in 13 countries”

The correct title is: “Measuring digital health literacy and its associations with determinants and health outcomes in 13 countries”

In the original published article the corresponding author‘s e-mail was diamos@zahav.net.il

The corrected e-mail is: dlevin-za@univ.haifa.ac.il

A correction has been made to **Abstract**, *Methods*

The term “HLS_19_-DIGI” has been added and the sentence below has been added:

“The instrument is a modified and extended version of the Digital Health Literacy Instrument (DHLI).”

So that the section now reads:

“Methods: Using a concept validation approach, the aim of the study was to validate the digital health literacy measure HLS_19_-DIGI, applied in the European Health Literacy Survey (2019–2021) of the WHO M-POHL network, analyzing data from 28,057 respondents from 13 countries. The instrument is a modified and extended version of the Digital Health Literacy Instrument (DHLI).”

In **1. Introduction**, *1.1 Existing research and measures of digital health literacy*, paragraph 3, “(DHLI)” has been changed to “(Digital Health Literacy Instrument- DHLI)”.

In **1. Introduction**, *1.2 Rational for a further developed digital health literacy measure*, the header “Rational for developing a new digital health literacy measure” has been changed to “Rational for a further developed digital health literacy measure”.

In **1. Introduction**, *1.2 Rational for a further developed digital health literacy measure*, paragraph 2, “opportunity to develop and validate a new measure for DHL“ has been changed to “opportunity to further develop and validate a further developed measure for DHL.”

In **1. Introduction**, *1.2 Rational for a further developed digital health literacy measure*, paragraph 3, “This article is part of a series of papers, introducing new health literacy tools that have been developed, applied, and tested through the HLS_19_ study (27–30)” has been changed to ”This article is part of a series of papers, introducing health literacy tools that have been developed, applied and tested through the HLS_19_ study (27-30)”.

In **1. Introduction**, *1.2 Rational for a further developed digital health literacy measure*, paragraph 3, “The aim of this series is to use the data collected in HLS_19_ to examine the psychometric properties of the newly developed health literacy tools and different aspects of their validity” has changed to “The aim of this series is to use the data collected in the HLS_19_ to examine the psychometric properties of the newly or further developed health literacy tools and different aspects of their validity”.

In **2. Materials and methods**, *2.1 The HLS*_19_*-DIGI instrument*, the header has changed from “2.1 Development of the HLS_19_-DIGI instrument” to “2.1 The HLS_19_-DIGI instrument”.

In **2. Materials and methods**, *2.1 The HLS*_19_*-DIGI instrument*, paragraph 1, “The DHL instrument developed for the HLS_19_ survey, named HLS_19_-DIGI, is based on the DHLI measure (16) but aligned more strongly with the concept and model of general health literacy proposed by the HLS-EU study (27) and promoted by M-POHL” has changed to “The DHL instrument further developed for the HLS_19_ survey, named HLS_19_-DIGI, is based on the DHLI measure (16) but aligned more strongly with the concept and model of general health literacy proposed by the HLS-EU study (27), and promoted by M-POHL.

In **2. Materials and methods**, *2.1 The HLS*_19_*-DIGI instrument*, paragraph 1, the citation (16) has been added, so that the sentence reads “Compared to the DHLI (16), the HLS_19_-DIGI adds the dimension of understanding digitally accessed health information and eliminates the redundancy on the topic of applying health information.”

In **2. Materials and methods**, *2.1 The HLS*_19_*-DIGI instrument*, paragraph 3, the citation (16) has been added, so that the sentence reads “We have adapted, shortened and extended the content of the DHLI tool (16), translated it into several languages and tested it, and this paper reports on the psychometric testing.”

In **4. Discussion**, paragraph 2, “This article described the conceptual background, development, and validation of a new instrument to measure DHL at the population level and investigated its determinants and associations with health outcomes “ has changed to “This article described the conceptual background, development, and validation of a further developed instrument to measure DHL at the population level and investigated its determinants and associations with health outcomes.”

In **4. Discussion**, *4.1 Limitations*, “For some countries, the non-response rates for several items were markedly higher than for other HLS_19_ measures. this may be partly due to the fact that people cannot evaluate something they do not do in everyday life, namely if they do not use the Internet to search for health information.” has changed to “For some countries, the non-response rates for several items were markedly higher than for other HLS_19_ measures, which may be partly due to the fact that people cannot evaluate something they do not do in everyday life, namely if they do not use the Internet to search for health information.”

In **5. Conclusions and implications**, “A compact, conceptually sound new instrument to measure DHL was validated for 13 languages in 13 countries, showing acceptable psychometric properties.” Has changed to “A compact, conceptually sound instrument to measure DHL was validated for 13 languages in 13 countries, showing acceptable psychometric properties.”

The original version of this article has been updated.

