# Supplementary material for: Measuring digital health literacy and its associations with determinants and health outcomes in 13 countries
Source: Front Public Health. 2025 Mar 20;13:1472706. doi: 10.3389/fpubh.2025.1472706 (PMC11966570; doi:10.3389/fpubh.2025.1472706)
Supplement: Supplementary file 1 [file Table_1.docx]

Supplementary Table 1: Description of the sample

|  | **Raw sample size** | **Age (SD)** | **Female %** | **ISCED <= 2 %** |
| --- | --- | --- | --- | --- |
| **AT** | 2967 | 52.31 (16.43) | 55.85 % | 10.52 % |
| **BE** | 1000 | 47.82 (16.11) | 50.40 % | 3.04 % |
| **CH** | 2502 | 50.01 (17.69) | 51.22 % | 13.03 % |
| **CZ** | 1599 | 49.37 (17.33) | 52.16 % | 45.99 % |
| **DE** | 2143 | 51.76 (18.17) | 50.42 % | 9.49 % |
| **DK** | 3602 | 57.19 (15.59) | 56.14 % | 9.50 % |
| **FR** | 1000 | 46.48 (15.86) | 51.10 % | 3.50 % |
| **HU** | 1195 | 52.91 (17.10) | 52.22 % | 42.59 % |
| **IE** | 4487 | 49.26 (15.61) | 50.32 % | 11.68 % |
| **IL** | 1315 | 44.12 (15.83) | 51.18 % | 8.14 % |
| **NO** | 2855 | 45.13 (17.50) | 51.28 % | 8.84 % |
| **PT** | 1247 | 46.14 (16.69) | 51.56 % | 40.50 % |
| **SK** | 2145 | 47.60 (17.95) | 52.07 % | 18.40 % |

AT = Austria, BE = Belgium, CH = Switzerland, CZ = Czech Republic, DE = Germany, DK = Denmark, FR = France, HU = Hungary,

IE = Ireland, IL = Israel, NO = Norway, PT = Portugal, SK = Slovakia

[The numbers for the score and age are in the format “mean (standard deviation)”. These are raw, unweighted numbers.]
