# Supplementary material for: Measuring digital health literacy and its associations with determinants and health outcomes in 13 countries
Source: Front Public Health. 2025 Mar 20;13:1472706. doi: 10.3389/fpubh.2025.1472706 (PMC11966570; doi:10.3389/fpubh.2025.1472706)
Supplement: Supplementary file 2 [file Table_2.docx]

Supplementary Table 2: Deviation from the population mean d-type score of the HLS_19_-DIGI-HI for selected vulnerable groups, per country and mean for all countries (equally weighted).

|  | **AT** | **BE** | **CH** | **CZ** | **DE** | **DK** | **FR**  **(*)** | **HU** | **IE** | **IL** | **NO** | **PT** | **SK** | **Mean** |
| --- | --- | --- | --- | --- | --- | --- | --- | --- | --- | --- | --- | --- | --- | --- |
| Population | 70.1 | 54.9 | 55.7 | 59.3 | 41.8 | 67.2 | 53.9 | 71.8 | 62.1 | 62.7 | 78.7 | 74 | 60.3 | 62.5 |
| Age > 76 | -9.7 | -5.8 | -16 | -2 | -23 | -11.6 | - | -18.3 | -11.5 | -4.9 | -8.4 | 1.7 | -34.9 | -12.0 |
| Low education (ISCED-0,1) | -20.2 | 7.6 | -10.6 | 1.5 | -8.4 | -24.1 | 6.2 | -6 | -11.1 | -8.8 | 3.4 | -9.9 | -20.7 | -7.8 |
| Social status (< 4 on scale 1 –10) | -2.2 | -5.8 | -5.9 | -3.7 | -8.2 | -9.5 | -4.3 | -5.8 | -4.7 | -9.1 | -4.3 | -6 | -19 | -6.8 |
| Financial deprivation | -11.3 | -2.4 | -4.9 | -8 | -10.4 | -9.6 | -4.7 | -9.6 | -8.7 | -7.3 | -13.4 | -8.7 | -14.5 | -8.7 |
| Low self-perceived health | -8.3 | 0.4 | -9.1 | -4.4 | -23 | -8.9 | -11.9 | -9.8 | -10 | -9.2 | -9.6 | -21.5 | -23.3 | -11.4 |
| Long-term illness | -4.1 | -1.4 | -4.7 | -1.2 | -3.3 | -2.7 | -1.7 | -4.7 | -0.6 | -3 | -1.2 | -8.2 | -4.7 | -3.2 |
| Limitation due to health | -6.6 | -3.3 | -4.7 | -3 | -4.7 | -5.2 | -0.7 | -7.2 | -2.9 | -1.7 | -2.3 | -9.3 | -9.8 | -4.7 |
| Visits to a GP (> 6) | -4.6 | -2.7 | -7.7 | -1 | -8.9 | -9.5 | -3.3 | -8.4 | -6.1 | -2.5 | -3.2 | -12.2 | -19.2 | -6.9 |

AT = Austria, BE = Belgium, CH = Switzerland, CZ = Czech Republic, DE = Germany, DK=Denmark, FR = France, HU=Hungary, IE=Ireland, IL=Israel, NO=Norway, PT = Portugal, SK=Slovakia(*) France limited participation to those below the age of 76
