# Supplementary material for: Measuring digital health literacy and its associations with determinants and health outcomes in 13 countries
Source: Front Public Health. 2025 Mar 20;13:1472706. doi: 10.3389/fpubh.2025.1472706 (PMC11966570; doi:10.3389/fpubh.2025.1472706)
Supplement: Supplementary file 3 [file Table_3.docx]

Supplementary Table 3: Multivariable linear regression model (standardized coefficients (ß) and R²) for the influence of general health literacy and determinants on digital health literacy for each country and for all countries combined (equally weighted) based on d-type scores.

|  | **AT** | **BE** | **CH** | **CZ** | **DE** | **DK** | **FR** | **HU** | **IE** | **IL** | **NO** | **PT** | **SK** | **All** |
| --- | --- | --- | --- | --- | --- | --- | --- | --- | --- | --- | --- | --- | --- | --- |
| GEN-HL | **0.44** | **0.44** | **0.47** | **0.56** | **0.54** | **0.53** | **0.60** | **0.46** | **0.47** | **0.67** | **0.46** | **0.53** | **0.43** | **0.51** |
| Gender female | -0.02 | 0.01 | 0 | -0.02 | -0.01 | **0.04** | -0.02 | 0.05 | 0.02 | -0.01 | **0.05** | -0.06 | -0.01 | 0 |
| Age in years | **-0.16** | -0.06 | **-0.17** | **-0.08** | **-0.20** | **-0.21** | **-0.17** | **-0.11** | **-0.04** | **-0.10** | -0.03 | **-0.10** | **-0.15** | **-0.13** |
| Education | **0.06** | 0.03 | 0.02 | 0.01 | **0.09** | **0.12** | 0.03 | 0 | **0.05** | **0.07** | **0.09** | 0.01 | **0.17** | **0.06** |
| Level in society | 0.01 | 0.03 | 0.05 | 0.03 | **0.07** | **0.05** | 0.04 | -0.02 | 0.01 | 0.03 | 0.01 | -0.07 | 0.05 | **0.03** |
| Financial deprivation | **-0.06** | **-0.09** | -0.04 | -0.04 | 0 | 0.01 | 0.03 | **-0.15** | -0.03 | 0 | -0.03 | -0.07 | **-0.14** | **-0.06** |
|  |  |  |  |  |  |  |  |  |  |  |  |  |  |  |
| R^2^ | 0.25 | 0.21 | 0.27 | 0.33 | 0.41 | 0.38 | 0.39 | 0.29 | 0.25 | 0.46 | 0.24 | 0.31 | 0.38 | 0.33 |
| Valid Count | 2253 | 988 | 1901 | 1249 | 1735 | 3537 | 1000 | 892 | 3516 | 1149 | 2272 | 760 | 1624 |  |
| Total Count | 2967 | 1000 | 2502 | 1599 | 2143 | 3602 | 1000 | 1195 | 4487 | 1315 | 2855 | 1247 | 2145 |  |
| Coefficients with *p*-values lower than 0.01 in bold. Due to rounding the numbers to two significant decimals, zeros may represent a value in the range of -0.005 to +0.005.  GEN-HL-Index: from 0=minimal HL to 100=maximal HL.  Education by 9 ISCED levels. from 0 (lowest) to 8 (highest level).  Level in society from 1=lowest level to 10=highest level in society.  Financial deprivation: 4 categories. from no deprivation (0) to severe deprivation (100). | | | | | | | | | | | | | | |

AT = Austria, BE = Belgium, CH = Switzerland, CZ = Czech Republic, DE = Germany, DK = Denmark, FR = France, HU = Hungary, IE = Ireland, IL = Israel, NO = Norway, PT = Portugal, SK = Slovakia
