# Supplementary material for: Measuring digital health literacy and its associations with determinants and health outcomes in 13 countries
Source: Front Public Health. 2025 Mar 20;13:1472706. doi: 10.3389/fpubh.2025.1472706 (PMC11966570; doi:10.3389/fpubh.2025.1472706)
Supplement: Supplementary file 4 [file Table_4.docx]

Supplementary Table 4: Multivariable linear regression models (standardized coefficients (ß) and R²) for the influence of digital health literacy and determinants on health outcomes (self-perceived health, GP/family doctor visits and specialist consultations) for each country and for all countries combined (equally weighted).

|  | AT | BE | CH | CZ | DE | DK | FR | HU | IE | IL | NO | PT | SK | All |
| --- | --- | --- | --- | --- | --- | --- | --- | --- | --- | --- | --- | --- | --- | --- |
| **Self-perceived health** |  |  |  |  |  |  |  |  |  |  |  |  |  |  |
| DHL | **-0.11** | -0.06 | **-0.08** | -0.06 | **-0.14** | **-0.11** | **-0.12** | -0.03 | **-0.05** | **-0.10** | **-0.07** | -0.08 | **-0.09** | **-0.10** |
| Gender (female) | -0.03 | 0.04 | -0.03 | -0.02 | -0.03 | **-0.05** | -0.01 | 0.05 | -0.03 | -0.02 | 0.03 | **0.09** | 0.02 | -0.01 |
| Age | **0.25** | 0.07 | **0.21** | **0.34** | **0.38** | **0.08** | **0.19** | **0.30** | **0.11** | **0.29** | **0.16** | **0.30** | **0.38** | **0.24** |
| Education | **-0.06** | -0.08 | -0.04 | **-0.11** | -0.03 | -0.03 | 0.01 | -0.05 | **-0.08** | 0.02 | **-0.09** | -0.06 | -0.03 | **-0.03** |
| Social status | **-0.10** | **-0.29** | **-0.18** | **-0.15** | **-0.09** | **-0.15** | **-0.23** | **-0.14** | **-0.12** | **-0.17** | **-0.20** | **-0.13** | **-0.10** | **-0.17** |
| Financial deprivation | **0.15** | -0.04 | **0.16** | **0.16** | **0.15** | **0.20** | **0.13** | **0.20** | **0.20** | **0.13** | **0.16** | **0.17** | **0.14** | **0.14** |
| R^2^ | 0.14 | 0.11 | 0.14 | 0.23 | 0.25 | 0.12 | 0.16 | 0.22 | 0.10 | 0.16 | 0.12 | 0.23 | 0.26 | 0.15 |
| **GP/family doctor** |  |  |  |  |  |  |  |  |  |  |  |  |  |  |
| DHL | **-0.10** | -0.02 | -0.05 | -0.01 | **-0.08** | **-0.07** | -0.06 | **-0.09** | **-0.05** | -0.04 | **-0.06** | -0.06 | **-0.09** | **-0.08** |
| Gender (female) | **0.09** | **0.08** | 0.03 | 0.00 | **0.09** | **0.10** | 0.07 | **0.14** | **0.17** | **0.09** | **0.16** | **0.19** | **0.07** | **0.09** |
| Age | **0.22** | **0.16** | **0.18** | **0.17** | **0.30** | **0.07** | **0.17** | **0.25** | **0.11** | 0.04 | 0.05 | **0.12** | **0.22** | **0.18** |
| Education | -0.01 | **-0.18** | -0.03 | **-0.09** | -0.02 | -0.02 | -0.01 | -0.03 | -0.02 | -0.07 | **-0.09** | -0.03 | -0.03 | **-0.03** |
| Social status | 0.05 | -0.06 | **-0.08** | -0.04 | -0.05 | **-0.09** | -0.02 | 0.00 | **-0.05** | 0.02 | 0.00 | 0.00 | 0.03 | -0.01 |
| Financial deprivation | **0.10** | -0.01 | **0.07** | **0.10** | **0.09** | **0.06** | **0.12** | 0.06 | **0.10** | **0.14** | **0.09** | 0.07 | **0.17** | **0.08** |
| R^2^ | 0.10 | 0.09 | 0.06 | 0.06 | 0.14 | 0.04 | 0.06 | 0.11 | 0.06 | 0.04 | 0.05 | 0.07 | 0.13 | 0.06 |
| **Medical /surgical specialist** |  |  |  |  |  |  |  |  |  |  |  |  |  |  |
| DHL | -0.04 | 0.01 | -0.03 | -0.06 | -0.03 | -0.04 | -0.04 | 0.00 | 0.00 | -0.06 | -0.05 | -0.06 | **-0.08** | **-0.07** |
| Gender (female) | **0.22** | **0.14** | **0.08** | **0.15** | **0.10** | **0.06** | **0.16** | **0.10** | 0.03 | **0.09** | 0.03 | **0.16** | **0.10** | **0.10** |
| Age | **0.14** | **0.16** | **0.12** | **0.19** | **0.27** | **0.08** | **0.13** | 0.09 | **0.14** | **0.20** | **0.09** | **0.13** | **0.24** | **0.15** |
| Education | **0.09** | -0.04 | **0.10** | 0.07 | 0.06 | 0.04 | **0.09** | **0.14** | 0.01 | **0.09** | -0.02 | **0.17** | 0.05 | **0.02** |
| Social status | -0.02 | **-0.09** | **-0.08** | -0.04 | -0.06 | -0.01 | 0.07 | -0.01 | -0.03 | -0.03 | -0.02 | 0.02 | 0.01 | **-0.02** |
| Financial deprivation | **0.06** | 0.06 | **0.12** | 0.04 | **0.09** | **0.09** | **0.13** | **0.15** | **0.06** | **0.08** | 0.02 | 0.07 | 0.06 | **0.10** |
| R^2^ | 0.09 | 0.06 | 0.05 | 0.06 | 0.11 | 0.02 | 0.06 | 0.06 | 0.03 | 0.08 | 0.01 | 0.07 | 0.09 | 0.05 |

Coefficients with p-values lower than 0.01 in bold. Due to rounding the numbers to two significant decimals, zeros may represent a value in the range of -0.005 to +0.005. HL-DIGI-HI score: from 0=minimal HL to 100=maximal HL.

Education by 9 ISCED levels, from 0 (lowest) to 8 (highest level).

Self-perceived social status from 1=lowest level to 10=highest level in society.

Financial deprivation: 4 categories, from no deprivation (0) to severe deprivation (100).

AT = Austria, BE = Belgium, CH = Switzerland, CZ = Czech Republic, DE = Germany, DK = Denmark, FR = France, HU = Hungary,

IE = Ireland, IL = Israel, NO = Norway, PT = Portugal, SK = Slovakia
